# Descending Control in the Auditory System: A Perspective

**DOI:** 10.3389/fnins.2021.769192

**Published:** 2021-10-18

**Authors:** Nina Kraus

**Affiliations:** Departments of Communication Sciences, Neurobiology, Otolaryngology, Northwestern University, Evanston, IL, United States

**Keywords:** afferent, efferent, auditory, descending control, memory, sound, BEAMS

Once upon a time, I spent hours threading microelectrodes into auditory cortex neurons and counting spikes as the rabbits learned a tone-signaled task. Now, it is not news that cortical neurons change their tune when sound acquires relevance. But, it was still novel in the late 1970s and it was new to watch this plasticity unfold over time (Kraus and Disterhoft, 1982). It had, after all, only been a decade since the idea of a hard-wired brain was upended by observations of a remapped somatosensory cortex in monkeys (Paul et al., 1972).

In the intervening 40 years, response plasticity has been observed in nooks and crannies of the auditory pathway. Questions remain. What is the time course? How persistent are the changes? To what extent does the type of sound matter? The type of training? The nature of the task? The context? How does hearing loss alter plasticity? Or aging? How do other sensory systems contribute to auditory plasticity? What are the roles of primary and non-primary pathways? How do the afferent and efferent systems interact in effecting changes in response properties? How does response plasticity propagate throughout brain circuitry?

I recently proposed the BEAMS hypothesis (the dynamic auditory **B**rain, via **E**fferent influence, attains a new default **A**fferent state that represents **M**emory for **S**ound; Kraus, 2021a) to tackle another question: how is auditory memory reflected in neural response properties? Here, I expand on BEAMS and provide additional context to emphasize that, in my opinion, afferent processing pathways in the auditory system, via the mechanism of descending efferent control, indeed store our memory for sound.

A one-sentence theme of this special issue and of our knowledge of descending control in the auditory system could be “the hearing brain is vast.” The narrowly defined hierarchy of the classical auditory pathway is firmly in the dustbin of history. How we think about sound, how we feel about sound, the movements that accompany sound, and what we experience with our other senses play key roles in shaping our auditory infrastructure (Kraus and White-Schwoch, 2015)—what I have come to call the “sound mind” (Kraus, 2021b). Our experience with sound—lifelong, in-the-moment, and all points between—leaves a legacy on this massively interconnected auditory system courtesy of the efferent system. Control of our auditory infrastructure, not only throughout the auditory system itself but by non-auditory brain systems, points to the pervasive influence of hearing to the lives of all organisms.

What might hearing without descending control look like? This is purely anecdotal, but I think it is telling. My ongoing dialog with clinicians has provided opportunities to investigate individuals with unusual medical histories. Between these case studies and our research in impaired, typical, and expert listeners, I have seen thousands of speech-evoked frequency-following responses (FFR). The single biggest, sharpest, 10-out-of-10, A-plus knockout of a response came from an individual with bilateral cortical lesions. Without cortical influence (indeed, his cortical evoked responses were entirely absent), this individual's FFR provided a glimpse of what a raw, unmodulated afferent system, left to its own devices, might look like (White-Schwoch et al., 2019).

This superb example of a lack of descending control of auditory processing serves as a cautionary tale on two fronts. First and more prosaically, it proves that bigger is not always better. But, second and more importantly, it reminds us we should not take any measurement in the afferent stream at face value. The flash of color on the fMRI, the electrophysiological squiggle, the DPOAE-gram, all must be interpreted as the product of an array of influences, some far removed from the local site of recording.

The principal realm of descending control is, of course, the efferent system: how our auditory function is altered by fill-in-the-blank—our emotions, our experience, our training, our vision, our environment… I am one of the efferent system's biggest cheerleaders, having made a career of looking at how auditory processing is affected by training and experience. However, of late, I find myself becoming more and more a champion of the afferent auditory system. While the focus of this issue is the efferent system, we must not lose sight that one of its most important purposes is to influence afferent processing.

How descending influence is manifested may be surprising, as the cortical-lesion case demonstrated. But, however it manifests, the functioning of the afferent stream under efferent control, expressed in my BEAMS hypothesis, reflects our auditory memories. The lower (in terms of the peripheral-central axis) the altered response property is found, the longer-term and more persistent the memory. Our afferent auditory system retains traces of our sonic history: a shift in the default auditory processing state. That is, we can think of the afferent auditory system as a default-mode network for hearing.

One of the brain's most important jobs is prediction. Predictive coding postulates incoming information is compared to an internal template shaped by experience. If the template does not fit the incoming information, the template is adjusted accordingly (Carbajal and Malmierca, 2018; Denham and Winkler, 2020). The study of this constant dialog of efferent control and afferent processing, as exemplified in this special issue, brings us closer to solving the mystery of how these templates/memories/default modes are formed and persist—a yin and yang of *plasticity* and *stability*, eternal cordial adversaries.

There is a range of plasticity across the auditory system—a continuum between stability and mutability. And the timecourse over which the alteration of a given set-state can be achieved varies as well. Non-primary pathway neurons are inherently more flexible than primary areas (Kraus et al., 1994; Atiani et al., 2014). Relatively speaking, central areas alter their properties more readily and quickly than more peripheral areas as revealed by simultaneous recording from multiple sites during auditory learning. In ferrets, changes in non-primary auditory cortex neurons precede changes in primary auditory cortex (Elgueda et al., 2019). In humans, non-primary AC has a larger influence than primary AC on speech perception (Hamilton et al., 2021), and cortical changes precede midbrain changes as demonstrated by Skoe et al.'s contribution to this issue (Skoe et al., 2021).

Reciprocity characterizes the nature of descending and ascending pathways. Efferent control itself is mutable (for better or worse). For example, an active efferent cholinergic system confers protection against noise-induced cochlear neuropathy (Boero et al., 2018) and auditory experience seems to shield the cochlea from loud sounds (Brashears et al., 2003; Skoe and Powell, 2021). A greater understanding of these and other principles throughout the neuraxis is increasingly within our grasp as investigative techniques improve.

This special issue of *Frontiers in Neuroscience* brings us a handful of overviews of the current state of “how things work,” reinforcing the general principle of reorganization of primary centers following non-primary reorganization. It also features original research that adds to our accumulated knowledge base and provides new methods to enable increased granularity as we continue to investigate the wheres and whens and hows of auditory learning in the interactive efferent and afferent streams.

The sound mind is remarkably dynamic, adaptive, and evolving. Each of us—through experience with the sounds that matter most to us—has forged a unique sound processing foundation to format our own construction of the sonic world. Our experience with sound adjusts the playing field (the afferent system), via descending control by the efferent system, so that new sound experiences are evaluated in light of our history.

## Author Contributions

NK conceived, wrote, and approved the submitted manuscript.

## Conflict of Interest

The author declares that the research was conducted in the absence of any commercial or financial relationships that could be construed as a potential conflict of interest.

## Publisher's Note

All claims expressed in this article are solely those of the authors and do not necessarily represent those of their affiliated organizations, or those of the publisher, the editors and the reviewers. Any product that may be evaluated in this article, or claim that may be made by its manufacturer, is not guaranteed or endorsed by the publisher.
